# Dioscorides Phakas on the Detection of Iron in Blood, Urine and Sputum

**Published:** 1946

**Authors:** M. Nierenstein

**Affiliations:** Formerly Demonstrator, School of Tropical Medicine, Liverpool, and Reader in Biochemistry, University of Bristol


### DIOSCORIDES PHAKAS ON THE DETECTION OF IRON IN BLOOD, URINE AND SPUTUM.

BY

THE LATE M. NIERENSTEIN, D.SC.,

*Formerly Demonstrator, School of Tropical Medicine, Liverpool, and Reader in Biochemistry, University of Bristol.*

---

DURING our visit to St. Félix on the Orinoco, we—my three Venezuelan colleagues and I—were the guests of Dr. McPherson, a general practitioner at St. Félix. His family, Peruvian by origin, had lived in St. Félix for several generations, and he and his father had studied medicine at the ancient Venezuelan University of Merída. Dr. McPherson senior had been interested in the history of medicine and had built up a good general practice. His son, our host, had developed the practice and had also become the Senior Physician of the State Hospital at Upatá, and thus held a well-paid appointment. As he had neither time for nor interest in the history of medicine, he presented to me a bundle of manuscripts, amongst which I found a Latin translation of the twenty-four books by Dioscorides Phakas on medicine, lost since about A.D. 1000. The late Dr. Walter Crum, of Bath, was of opinion that it was written in a seventeenth-century hand. It was copied by Father Jacobus from the original Latin MS. in one of the libraries at Salamanca.

It is well to realise that there were at least three physicians who wrote under the name Dioscorides, namely : Dioscorides Phakas, who was Cleopatra's physician ; Dioscorides Pedanios, the author of the famous *Materia Medica*, a contemporary of Nero, Vespasian and Pliny the Elder ; and there was also a third Dioscorides, generally known as The Younger, who lived at the time when Hadrian was the Roman Emperor. This Dioscorides made a Latin translation of some of the works of Hippocrates, which was severely criticied by Galen.

Nothing whatever is known as regards Dioscorides, called Phakas because of his freckles, which were distributed like pease (*phakoi*) all over his face. Hirsch[1] translates *phakoi* as " warts," which does not appeal to me : he says that Dioscorides Phakas was a follower of Herophilus who lived about B.C. 300., that his works on medicine were highly praised by Galen (*c.* A.D. 130-200), and that Suidas, who lived about A.D. 970,[2] mentions Dioscorides's works as extant in his exicon. Favrier[3] states that Menodotus, who wrote in the second century A.D., speaks of the works of Dioscorides Phakas with much respect.

Dr. McPherson senior, in his notes on the manuscript, says that he had found much evidence in Rhazes showing that the latter had made use of Dioscorides Phakas's books on medicine. Historically this is quite probable, as according to Campbell,[4] Rhazes lived *circa* A.D. 841-926, and thus had access to the original Greek manuscript. Le Clerc[5] and Neuberg[6] have already pointed out that Arabic writings are often the best depositories of lost Greek as well as Roman knowledge of medicine. Otherwise all other references to Dioscorides Phakas[7] can be traced to Haeser,[8] who, as will be seen, quite erroneously writes : " After the expulsion of all scholars from Alexandria by Ptolemy Phykon, Laodicea in Syria became the head-quarters of the herophilic school. Some well-known physicians learned the art of medicine at Laodicea : namely, Zeuxis, Alexander Philaletus, Heraclides, Dioscorides Phakas . . . "

In his preface Dioscorides gives a brief sketch of his life, and he states that he had studied medicine at Alexandria under Zsosimos.[9] His fellow-students, alphabetically arranged, were Alcamaceonus, Blossius, Claudius, Empedocles, Ephorisius, Epicharmus, Eponymus, Fabius, Hegisippus, Heraclitus, Mercisteus, Pytheus and Xenophanus, all of whom seem to have disappeared into the oblivion of general practice. These names, however, go to show that at the time of Dioscorides, Alexandria still had a flourishing school of medicine, in spite of the bad influence on the city of Ptolemy Phykon. On qualifying, Dioscorides became the assistant of Zsosimos for several years, and he spent altogether ten years in Alexandria. He made the sanatoria his special study, and after having visited several of them he spent over a year at the sanatorium at Epidaurus, of which he writes : " I was much impressed by the good work they did at the Epidaurus Sanatorium, but still more by the sound finance of the place. I have found in Epidaurus what my Queen sent me to find."

Dioscorides was the nephew of Ptolemy XIII and thus a cousin of Cleopatra. The Ptolemaic dynasty was Greek, and his name Dioscorides was obviously not assumed, as was frequently the case with Roman physicians and surgeons. As regards his nickname, Phakas says that it was given him by his fellow students and that Zsosimos often playfully used to call him by this name. He mentions that Zsosimos, during his lectures, when he referred to the opinion held by the ancient Egyptians, but not accepted by Zsosimos, namely, that all that is white in the body is contributed by the father and all that is coloured by the mother, used to say : " Now look at our young friend Phakas, do you expect that any self-respecting mother would sprinkle her child with freckles ? " To which he adds that " this joke used to amuse the others, but I did not care for it."

Until the age of forty-three, Dioscorides held different court appointments, mainly in the Treasury. His experiences there are reflected throughout his medical works dealing with metabolism, and account also for his dislike of all Aristotelian inexactitudes. Of Aristotle he says : " It would have been a good thing if he had only given his mind to philosophy and avoided medicine."

Dioscorides was in his fifty-seventh year when he returned to Egypt, where he found Cleopatra deep in her intrigues with Rome. Dioscorides's latent xenophobia became acute, and in all his writings he talks of the Romans in Egypt as "those barbarians from the north." How long he was in the service of Cleopatra is difficult to say, but it is doubtful that he survived Cleopatra when she died in 30 B.C.

In a previous communication[10] I traced the early history of the first chemical reagent, namely, Pliny's test for the detection of iron in verdigris,[11] in which Pliny used papyrus soaked in aqueous extracts of galls.[12] Later on I suggested that these observations of Pliny were probably of much older standing and may have originated from the Greek oracles.[13] Oak-leaves soaked in the holy springs[14] would when dry give hieroglyphics which could pass for Greek characters, providing that these springs contained iron, as the veins of oak leaves are rich in tannin. Since then I have had the opportunity of consulting Gruber[15] and found that nearly all the waters in Greece contain iron. Thanks to Dr. Wallis, the Deputy Director of the Bristol Museum, who gave me ancient and modern pieces of papyrus, I was able to confirm Pliny's statement. From the literature at my disposal[16] it was evident that Pliny wrote his *Historia Naturalis* in A.D. 60, which thus dated Pliny's reagent. I further pointed out that Pliny's test is still officially in use in Venezuela[17] and that the vinegar industry[18] in this country still uses it. I now find that it is also the official method in Mexico[19] and that it was used in 1807 as a microscopical test for the detection of tannin[20] in plant-anatomy.

It was this test Dioscorides used a hundred years before Pliny for the detection of iron in blood, urine and sputum, after incineration and extracting both the ash and the galls with rainwater—" heavenly water," as he called it. To understand why Dioscorides made these investigations, we have to realise that his training was Egypto-Alexandrian with a strong Greek bias, which taught that blood from different parts of the human body differs[21] and also that the blood of a man differs from that of animals.[22]

By using this test he convinced himself that this theory is incorrect and then turned to human pathology ; he examined blood-containing sputum in cases of what was probably phthisis, and what he called " bloody urine." He writes as follows : " On one occasion I was preparing a concentrate of goat-blood for one of the ladies of the court who feared that she was pregnant, a great disability, as Cleopatra objects to pregnant ladies at court. I then had a message from the Queen and after several hours' absence I found that the blood had evaporated to dryness and burned to cinders. I then examined it in the manner described and found that it contained iron. I thus for the first time convinced myself that the goat's blood contained iron. I then turned to human blood and diseases, namely sputum that contained blood, and ‘ bloody urine.’ I found that they all contained iron, and I felt convinced that the belief that the blood of mankind differs from that of the animals is wrong.''

Later on he wrote and dated his entry for the first and only time. “ To-day my Queen was twenty-five years old [46 B.C.], and I have made a discovery which I find difficult to understand. Several years after I had found iron in ‘ bloody urine,’ I used to leave this urine standing in narrow wine-glasses, but there seemed to be little change in these urines ; apparently I was too hasty in wanting to find the truth. One day, for some reason I have forgotten, probably the fact that I was sent for by the Queen, as she always wanted us, whom she trusted, to be present on her birthday, the day the gods sent her to Egypt. Several days later I found that it looked like healthy urine and that it had a divine golden colour. This made me think for many days, because on using papyrus treated with gall-extract in the manner described I found that it contained iron. What it means I do not know. This is a secret only the gods know. Since then I have repeated this as we have much ‘ bloody urine ’ in Egypt both in men and women.''

It would be rash to say that Dioscorides thus described blackwater fever, as there is no evidence that Egypt in ancient days had either blackwater fever[23] or even malaria.[24] However, Dioscorides's observations are suggestive enough to be compared with the present-day data given by Dukes[25] for the examination of blackwater urines.

Dukes recommends the following procedure for the diagnosis of blackwater fever. Test-tubes filled with urine are observed on standing. On the first day the urine is black and contains methaemoglobin and oxyhaemoglobin. On the second day the urine is dark red, it is in the stage of devervescence, and oxyhaemoglobin predominates. On the third day the urine is brownish and recovery has set in, as there are only traces of methaemoglobin present. Table III by Dukes shows these details very clearly.

Quoting Fairley and Bromfield (1934), Dukes writes that they “ consider the haemolytic agent in blackwater fever to be dependent on some metabolic breakdown precipitated by the administration of quinine . . . . The blood corpuscles are first haemolysed and the liberated haemoglobin subsequently converted into methaemoglobin and other pigments which are excreted in the urine.'' Their observations seem to agree with those made by me[26] where it was found that out of thirteen blackwater urines twelve contained a disintegration product of quinine which had haemolytic properties and was provisionally named Haemoquinic Acid. However, all malaria urines contained haemoquinic acid after the administration of quinine, with the difference that in cases of blackwater the haemoquinic acid content is 578 times greater than in normal malaria cases. These results lead, in my opinion, to the conclusion that blackwater fever is due to idiosyncrasy in malaria-infected individuals.

Even if one assumes that Egypt in the time of Dioscorides had malaria and thus also blackwater fever, the production of haemoquinic acid from quinine cannot account for his " bloody urine," as quinine was not known until the discovery of America.[27] Dioscorides, in his book on fevers, to be dealt with in a later essay, says : " We have all kinds of fevers ; some we know how to treat and of some we have no knowledge whatever. There is a fever which none of us, including our priests, understands, and we do not know how to treat it, unless we use the extract of a herb which comes from far away. Early in the summer of every fourth year a large caravan of 500 to 1,000 camels leaves Egypt, and they are away for three years, when they bring many sacks of this herb, and we all hunger for it. They bring enough to last three years. I have talked to the men and they tell me that it takes a year to get to the place where the plant grows, as they have to climb many mountains. It takes a year to collect the herbs and a year to come back, as many of the camels are lost and a caravan of 1,000 camels is generally reduced to about 700. Also many of the men are killed by the natives of that country, who speak a foreign language, have yellow faces and very little hair on their faces and other parts of their body. That is all I have been able to find out as regards the country from where the herb comes."[28]

[In conclusion I mention that Dr. McPherson desired that the MSS. be presented to me and should be known as the St. Félix Manuscripts.]

### REFERENCES AND ANNOTATIONS.

1 Hirsch : *Biographisches Lexikon der hervorragenden Aerzte. . . .* (6 vols.) Wien and Leipzig, 1885-1888.

2 Seyffert : *A Dictionary of Classical Antiquities.* Revised and edited with additions by Nettleship and Sandys. London, 1894.

3 Favier : *Un Médecin Grec du II-ième Siècle A.D.* Paris, 1926.

4 Campbell : *Arabian Medicine . . . .* (2 vols.) London, 1926.

5 Le Clerc : *Histoire de la Médecine arabe* (2 vols.) Paris, 1876.

6 Neuberg : *Geschichte der Medizin.* Stuttgart, 1908.

7 Haeser : *Lehrbuch der Geschichte der Medicin* (3 vols.), Jena, 1875-1882 ; and *Geschichte der Chirurgie . . . ,* Jena, 1864.

Gurlt : *Geschichte der Chirurgie . . .* (3 vols.) Berlin, 1898.

This error does not occur in Sprengel : *Versuch einer pragmatischen Geschichte der Arzneikunst* (5 vols.), Halle, 1792-1828 ; and has also not been perpetrated by Allbutt : *The Historical Relations of Medicine and Surgery,* London, 1905 ; and *Greek Medicine in Rome,* London, 1921.

8 Haeser : *Grundiss der Geschichte der Medizin.* Jena, 1884.

[9] Zsosimos was a common name and it is therefore difficult to decide who this Zsosimos was. I am, however, inclined to think that he was the Zsosimos who wrote the famous letters to Theosobia, whom from time to time he called " my Queen, Beloved, Sister or Wife," which dealt amongst other things with fermentation ; *see* Berthollet : *Introduction à l'Étude de Chimie des Anciens et du Moyen Age*, Paris, 1889 ; and *Les Origines de l'Alchime*, Paris, 1885 ; Kopp : *Beiträge zur Geschichte der Chemie*, 1869 ; Braunschweig : *Die Alchemie in älterer und neuren Zeit* (2 vols.), Heidelberg, 1886 ; Hopkins : *Alchemie, Child of Greek Philosophie*, Harvard, 1934. Like Zsosimos, Dioscorides is also interested in fermentation, which he applies to digestion (to be dealt with more fully in a subsequent essay giving Dioscorides's work on digestion as compared with Zsosimos's researches on the fermentation of wine and other beverages).

[10] Nierenstein : *Isis*, xvi. 439, 1931.

[11] The origin of the term " verdigris " has been the object of much philologica speculation, and it seems to be a corruption of the Anglo-French *Vert de Grèce* (Greek Green) ; *see* Bailey and Bailey : *An Etymological Dictionary of Chemistry and Mineralogy*, London, 1939. Similarly the German *Spanisch Grün* for " Greek Green " is of the same philological origin ; *see* Beckmann : *Beiträge zur Geschichte der Erfindungen* (5 vols.), Leipzig, 1786-1806 ; who points out that it was also known according to Maader : *Teutsche Sprach oder Dictionarium germano-latinum*, Zürich, 1561, as *Spanisch Grün*, which he translates as *viride hispanicum*. Beckmann suggests other origins, and reference should be made to him for further study on the philology of " verdigris."

[12] Pliny : *Historia Naturalis*, xxxiv. 2 ; *see also* Bailey : *The Elder Pliny's Chapters on Chemical Subjects* (2 vols.), London, 1932.

[13] Nierenstein : *The Natural Organic Tannins*. London, 1934.

[14] Gallaeus : *Sibylina Oracula . . .* Amstelodamii, 1789. The large preface by Gallaeus is in itself a monograph of the different Greek as well as Roman modes of divining, and makes most interesting reading.

[15] Gruber : *Die Quellen Griechenlands chymisch, physisch und medizinisch Untersucht* (3 vols.), Weisskirchen, 1756-1773.

[16] Nierenstein : *Analyst*, lxviii. 212. 1943.

[17] *Farmacopea Venozolana*. Carácas, 1928.

[18] Mitchell : *Vinegar : its Manufacture and Examination*. London, 1926.

[19] *Farmacopea Mexicana*. Mexíco, 1937.

[20] Baker : *The Discovery of the uses of Colouring Agents in biological Micro-technique*. London, 1945.

[21] Hebrew medicine probably influenced Egyptian medicine. According to Hebrew medicine the blood of man is holy, whereas that of animals is not pure; *see* Ebstein : *Die Medizin im Neuem Testament und im Talmud*, Stuttgart, 1903 ; Brim : *Medicine in the Bible*, New York, 1936.

[22] For the teaching of the Alexandrian school of medicine consult the easily accessible works of Allbutt, especially his *Greek Medicine in Rome*, where on page 308, referring to Galen's theory that the veins have their roots in the liver, Allbutt adds : " The Chinese to this day, I am told, hold that the right pulse tells the state of the liver, and the left the state of the heart." *See also* Walsh : *Old-Time Makers of Medicine*, New York, 1911. There is no doubt whatever that there was a strong eastern influence on Greek and Egyptian medicine, as evident from Preuss : *Biblisch-talmudische Medizin*, Berlin, 1911 ; Budge : *The Syriac Books of Medicine* (2 vols.), Oxford, 1913 ; Breasted : Introduction in vol. i. of *The Edwin Smith Surgical Papyrus*, Chicago, 1930.

[23] Scott : *History of Tropical Medicine* (2 vols.), London, 1942.

[24] Brim : *Medicine in the Bible . . .*, New York, 1936 ; which I found, however, not to be reliable in many respects, as the author is far too enthusiastic to prove his points. Brim seems not to have been aware of Preuss : *Biblisch-talmudische Medizin*, Berlin, 1911—a remarkably unbiased work. I must confess that I find it very difficult to take seriously Dr. Brim, who speaks of a Mosaic Health Board with " Moses as President and Aaron as Vice-President."

25 Dukes : *Urine Examination and Clinical Interpretation*, London, 1939.

26 Nierenstein : *Jour. Royal Army Medical Corps.*, xxxii, 218, 1919 ; and for the extension of this work see Nierenstein in *Observations on Malaria*, edited by Ross, p.74, London, 1919.

27 The story of the cinchona bark has been told by many, but I do not know of any more fascinating historical review than that by Flückiger and Hanbury : *Pharmacographia*, pp. 341-348, London, 1879. They make it clear that from a statement by Humboldt (1807) : " the Peruvians (Mexicans) would rather die than have recourse to what they consider so dangerous a remedy." I have spent some considerable time amongst the Caroni Indians, who live on the Caroni River, a tributary of the Orinoco, and I hope one day to write more fully of their pharmacy and medicine. I mention, however, that the Caronis, who were fugitives from Mexico long before the Spanish Conquest, still refuse to take quinine, especially the older people. They use the extract of a plant they call *tzompo pachtzi*, identified as *Erythrina corallorida D.C.* by Emmart : *The Badanius Manuscript . . .*, p. 283, Baltimore, 1940. The Caronis know blackwater fever in men and women, and they look upon it as a form of menstruation in both sexes.

28 The information contained in Dioscorides is too fragmentary to allow any conclusion, but one cannot help but wonder if it does not refer to what is known as *Ch'ang Shan* (*Dichroa febrifuga*), described as an antimalarial by Chang-Shaw Jang : *Chinese Medical Journal*, lxii. 185, 1944. Dichroa, like cinchona, may also be the cause of blackwater in malaria. At the present time too little is known of this plant to allow certain deductions ; however, Dioscorides's description of the method by which it was imported is suggestive. In this connection it is interesting to note that Meyer : *Botanische Erläuterungen zu Strabons Geographie . . .*, p.127, Königsberg, 1852, mentions a species of Dichroa which according to Strabo (*c.* 63 B.C.-A.D. 19) was used as an internal antiseptic against fevers in North Africa.

Although at present it is best not to identify Dioscorides's "bloody urine" with blackwater fever, the clinical details of eight cases given by him remind one of the many cases quoted by Stephens: *Blackwater Fever*, Liverpool, 1937. Once historians are agreed, contrary to Scott,[23] that Hippocrates's fourteen cases of "bloody urine" are blackwater fever, Dioscorides becomes the second and Galen the third physician to have described the disease.

